# Optimization of Nano-Tangeretin Recrystallization via Natural Surfactants in the Antisolvent Precipitation Process: Physicochemical Characterization and Antioxidant Activity

**DOI:** 10.3390/nano15110791

**Published:** 2025-05-24

**Authors:** Yan Huang, Wenxuan Huang, Xiaonan Zhang, Zhiwei Liu

**Affiliations:** Life Science College, Jiaying University, Meizhou 514000, China; yanhuang@jyu.edu.cn (Y.H.); 202101260@jyu.edu.cn (W.H.)

**Keywords:** natural surfactants, nano-tangeretin in particles, solvent–antisolvent precipitation, antioxidant activity

## Abstract

In this study, an improved method combining natural surfactants with a solvent–antisolvent precipitation technique was developed to prepare highly effective nano-sized tangeretin particles. Various natural surfactants were tested and compared, and the formulation was optimized using Plackett–Burman and Box–Behnken design methodologies. The optimal preparation conditions were identified as follows: a tangeretin–dimethyl sulfoxide (DMSO) solution concentration of 5.23 mg/mL, surfactant concentration of 4.72%, and a rotor diameter of 20 mm. Under these conditions, uniform nano-tangeretin particles with an average size of 428.73 ± 30.25 nm were successfully produced. The preparation process significantly reduced particle size without chemical structure of tangeretin, as confirmed by spectral analysis. Importantly, the free radical scavenging activity of the nano-tangeretin was markedly enhanced, showing 65.4% increase in DPPH radical inhibition compared to the unprocessed powder. These results demonstrate that the proposed method can improve the bioactivity and dispersibility of tangeretin, providing a valuable strategy for the efficient utilization and industrial-scale production of bioactive compounds from natural resources.

## 1. Introduction

Surfactant chemical synthesis during industrial production is not conducive to clean production because residual components may be attached to the compound’s surface and cannot be broken down, causing toxic and adverse effects on the environment, ecosystem, and even human health [1,2,3]. Synthetic surfactants have a poor economy, limited recovery performance, and high manufacturing cost concurrently [4]. Consequently, there is a growing need for natural surfactants, particularly in the production of food and pharmaceuticals. Applications of natural surfactants have been reported, for example, Zhang et al. used natural surfactants to extract and separate glycoside A and polysaccharide from lily [5]. Wang et al. used saponins as surfactants to separate and extract phenolic acid, coumarin, phenylpropyl, and other hydrophilic phytochemicals from acanthopanax stems [6], but thus far, there has been no research on the application of natural surfactants in the processing field of solvent–antisolvent and ultra-micro technology.

Valentina et al. prepared nimesulide with hydrophilic polymer by solvent–antisolvent method, and successfully enhanced the dissolution rate and bioavailability of the prepared sample [7]. It is a new technology that combines jet technology, homogenization technology, and grinding technology. Additionally, the solution system’s treated nanoparticles do not cause dust pollution and are safe for the environment [8]. There are two benefits to using surfactants in solvent–antisolvent precipitation techniques: first, the surfactant has an amphiphilic structure, allowing for the reduction in surface tension in solution systems; the compound is soluble and permeable, allowing for the formation of nanoscale micelles, which are uniformly sized and small nanoparticles [9]. Second, the surfactant can enhance the antisolvent’s effectiveness and the powder’s rate of recovery. The different shapes and particle sizes of the compound will have a partial effect on water solubility [10], but the polarity of the compound, including the presence of carboxyl and hydroxyl groups on its surface and inside, will have a partial effect on water solubility. For example, the water solubility of sugar is dependent on the hydrophilic groups on its surface. It is advantageous to expose the hydrophilic group and improve its water solubility when the particle size of the functional component is decreased.

Tangeretin is a naturally occurring methoxy-flavonoid molecule that is highly sought after for its anti-fatigue, antioxidation, and anti-inflammatory capabilities in the realms of food chemistry and medicinal applications. Due to the structure of the raw tangeretin powder, which determines its poor water solubility and low bioavailability, its application has been restricted. Such large particles with poor solubility result in limited organism uptake and metabolism, in addition to producing medication waste during application and potentially harmful side effects on humans. Natural substances with significant surfactant applications include alkyl glycosides, sucrose fatty acid ester, peanut protein isolate, tea saponin, and peanut protein isolate [11,12,13,14]. These naturally isolated surfactants are less hazardous, more ecologically and biologically friendly, and better for the environment when compared to chemically manufactured surfactants for food processing applications [15].

We therefore chose tea saponin, peanut protein isolate, sucrose fatty acid ester, and C8-alkyl glucoside as additives. The difference between nano-tangeretin particles (TUP) and raw tangeretin powder (RTP) was compared by preliminary evaluation of antioxidant properties, which provided a scientific basis for the actual clean production of tangeretin and the development of natural antioxidants.

## 2. Materials and Methods

### 2.1. Materials

Tangeretin (purity ≥98%) was obtained from Shanghai Yuanye Co., LTD. (Shanghai, China). Tea saponin (purity ≥98%), peanut isolate protein (purity ≥98%), sucrose fatty acid ester (purity 98%), and C8-alkyl glucoside (purity 98%) were purchased from Shanghai Yuanye Co., LTD. (Shanghai, China). Dimethyl sulfoxide (DMSO, purity ≥99%); 2,2-diphenyl-1-picrohydrazyl (purity 98%); 2,2-diazo-bis (3-ethylbenzothiazol-6-sulfonic acid) (purity ≥98.5%); 2,6-di-tert-butylp-cresol, purity ≥98%; sodium hydroxide (purity ≥99%); sodium dihydrogen phosphate; phosphoric acid; and potassium persulfate were purchased from Tianjin Tianli Co., Ltd. (Tianjin, China).

An ultrasonic cleaner (KJ-1340AL) was purchased from Shenzhen Kejie Co., Ltd. (Shenzhen, China), a vacuum freeze dryer (JF-10 N-50A), was purchased from Shengwen Instrument Technology Co., Ltd. (Hangzhou, China). The nozzle (Hefang Co., Ltd., Tianjin, China) has a flat head shape (stainless steel 304). The outer diameter is 6 mm, the wall thickness is 2 mm, and the inner dimensions are between 200 and 600 μm. The nozzle length is 20 mm.

### 2.2. Preparation of Nano-Tangeretin Particles

**Figure 1:**
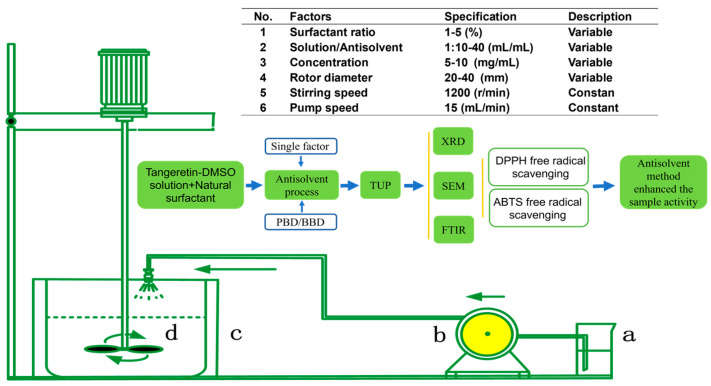
Flowchart and parameters of the APTUP process (a: solution; b: transfer pump; c: reaction vessel; d: Stirring paddle).

In this experiment, the solvent was dimethyl sulfoxide (DMSO), the antisolvent was deionized water, and the tangeretin solution was made at room temperature (25 °C). In the reaction tank, 100 mL of surfactant aqueous solution with a concentration of 1% was created first, followed by tangeretin solution with a concentration of 5 mg/mL. The tangeretin solution was sprayed into the reaction tank by a vacuum peristaltic pump (Model: GM-0.5B, Shenzhen, China) with a flow range of 1–30 mL/min. The homogenization rate was 1200 r/min, and the flow rate was 15 mL/min. Tangeretin nanocrystalline nuclei precipitated instantly, and the mixed solution was filtered through a 0.8 μm filter membrane and vacuum freeze-dried for 24 h to yield dry TUP. The specific experimental process and parameters are shown in Figure 1.

### 2.3. Surfactant Screening

During the experiment, surfactants aided in the creation of tiny molecules, and the effects of a number of distinct natural surfactants (A-sucrose fatty acid ester, B-tea saponin, C-peanut protein isolate, and D-alkyl glycoside) on the size of ultrafine powder were explored. In each experiment, 1–5% surfactant was added into tangeretin solution, with the volume ratio of solution to antisolvent being 1:10–40 mL/mL, the diameter of the rotor being 10–40 mm, and the stirring speed being constant at 1200 r/min. The antisolvent and natural surfactant are completely dissolved in the homogenate state, and TUP is produced during the homogenization and jet operations.

### 2.4. Response Surface Plackett–Burman Design Experiment

The preliminary experiment results revealed that five factors and their variables had the most significant influence on the size of TUP during the preparation of APTUP, which were A (Homogenate speed-r/min), B (Surfactant ratio-%), C (Rotor diameter-mm), D (Solution concentration-mg/mL), and E (Liquid–liquid ratio-mL/mL). The Plackett–Burman design (PBD) of the response surface is an optimization strategy for reducing the number of dimensions. Therefore, in this procedure, we attempted to utilize PBD to analyze the following experimental findings in the production of APTUP, which consisted of 12 run trials, each was run three times with varied combinations of 5 independent variables at high (+) or low (−) levels.

### 2.5. Response Surface Box–Behnken Design to Optimize Nano-Tangeretin Particles Size

Two stages of optimization were carried out. Box–Behnken design (BBD) was utilized to screen critical parameters using PBD. The optimization was performed in two stages: key parameters were screened using PBD, and then the resulting PBD parameters were further optimized using Box–Behnken design (BBD). The maximum and minimum (+1, −1) levels of the chosen parameters were determined by preliminary experiments carried out prior to the PBD experiment. Important influencing factors were chosen through PBD, and the parameter range of the factors chosen in BBD was in line with that of PBD. The average particle size of TUP was used as the response evaluation of the dependent variable (Y) in the initial experimental analysis of PBD that was previously discussed. We then optimized the data using the Pareto diagram results and selected the most significant conditions to further optimize the response surface Box–Behnken design (BBD). Analysis of variance (ANOVA), mapping RSM, and regression analysis were utilized to look at the dependent variables’ process parameters. The RSM method, which was used to determine the ideal operating parameters, produced the best APTUP process results.

### 2.6. Microstructure of Powders

The microstructure and distribution of particle sizes in RTP and TUP were examined using a scanning electron microscope which manufactured by Hitachi High-Technologies Corporation (Hitachi jsm-7500 F, Osaka, Japan) and a fluorescence microscope was manufactured by Carl Zeiss Microscopy GmbH, Jena, Germany (Axioscope-5, Berlin, Germany), respectively. A uniform and a thin conductive coating were created, the double-sided copper conductive adhesive glued to the sample table, the test sample powder fixed on the tray, and a small amount of the tangeretin sample glued to the double-sided copper conductive adhesive. Finally, a thin layer of metal ultrafine particles was sprayed on the prepared sample. Following gold spraying, the samples were observed at currents of 50 mA, time of 30 s, and current of 3 mA. SEM images were then taken at accelerated voltages of 15 kV and 20 kV, respectively, and the best image quality of the samples was taken, saved as a screenshot for future reference, and analyzed.

### 2.7. The Method of Infrared Spectral Analysis

We precisely weighed 2 mg of tangeretin sample powder and 190 mg of KBr, blended them entirely, dried the resulting mixture for two minutes in an infrared drying oven, ground it in the same direction, and then molded it into tablets. The findings of a study using infrared scans with wavelengths ranging from 400 to 5000 cm^−1^ were documented.

### 2.8. Crystal Properties of the Samples

For samples of tangeretin, an XRD powder diffractometer (Philips, Eindhoven, The Netherlands) was used to investigate the crystal structure. Tangeretin particles weighing 10 mg were equally dispensed on a glass slide before examination. Radiation of Cu K^−1^ was delivered at 30 mA and 40 kV. The XRD spectrum was recorded in 2θ, and the scanning range was 10–70°, with a scanning rate of 5°/min and a step size of 0.02°. According to Valenzuela and Rodriguez-Llamazares (2016) [16], the fundamental idea behind crystal region statistics is to first obtain a smooth line, then distinguish the crystalline and amorphous states in the X-ray diffraction pattern and finally determine crystallinity. Applying a moving average smoothing approach to the initial pattern allows for the creation of this smooth line. We used the Bruckner technique to compute the crystallinity of XRD; the crystallinity of XRD was calculated using R-studio (version: 0.98.507), and the results are discussed by drawing a table.

### 2.9. Thermal Analysis of Tangeretin Samples

A DISCOVERY DSC250 differential scanning calorimeter was manufactured by TA Instruments (New Castle, DE, USA) was used to assess the heat absorption of the tangeretin samples. Each tangeretin sample was placed in an aluminum pan with approximately 5 mg. Next, it is heated at a rate of 10 °C each minute, ranging from 25 to 350 °C.

### 2.10. Determination of Antioxidant Capacity

#### 2.10.1. Preparation of Sample Solution

RTP and TUP were dissolved in deionized water at a concentration of 10 mg/mL, and a solution of 2,6-di-tert-butylp-cresol (BHT) was employed as a positive control at the same concentration. The three samples underwent a 10 min ultrasonic treatment at 300 W, followed by a 10 min centrifugation at 12,000× *g* r/min. The supernatant was then diluted to the desired concentration range for measurement. Utilizing a UV spectrophotometer, the absorption value was calculated and statistical information was gathered.

#### 2.10.2. DPPH/ABTS Free Radical Scavenging Experiment

The exact weight of 3.84 mg of DPPH powder was used to make a 0.1 mmol/L DPPH-anhydrous ethanol solution, which was then added to 100 mL of anhydrous ethanol. Deionized aqueous solution (Blank control), DPPH-anhydrous ethanol solution, and samples of all tested liquid concentrations were mixed uniformly in a 1:1 ratio. The absorbance was measured using a UV spectrophotometer with a 517 nm wavelength following the blackout reaction for 30 min. As a positive control, a BHT solution with the same concentration gradient was utilized. The DPPH free radical scavenging rate calculation formula, which reads as follows, was used to express the antioxidant capacity:

SC_1_ (%) = (A_0_ − A_1_)/A_0_ × 100% (1)

A_0_ is the absorbance measured at 517 nm in the blank control group, whereas A_1_ is the absorbance obtained at 517 nm in the sample solution under investigation. SC_1_ is the clearance rate of the DPPH free radical at this wavelength. Deionized water is mixed with ABTS and K_2_S_2_O_8_ in a certain ratio. Potassium persulfate aqueous solution (4.9 mmol/L) and ABTS aqueous solution (7 mmol/L) were combined in a 1:1 ratio to create ABTS mother liquor, which was kept at 4 °C for 12–16 h while being shaded. To create an ABTS solution for usage, it is diluted 20 times with a phosphate buffer that has a pH of 7.4.

Deionized aqueous solution (Blank control) and samples of various tested liquid concentrations were combined uniformly with ABTS solution at a ratio of 1:9 before the light was turned off for 30 min. A 734 nm UV spectrophotometer was used to measure the absorption. The positive control was a BHT solution with the same gradient of concentrations. The ABTS free radical scavenging rate calculation formula, which reads as follows, was used to express the antioxidant capacity:

SC_2_ (%) = (A_2_ − A_3_)/A_2_ × 100% (2)

SC_2_ is the scavenging rate of ABTS free radicals at 734 nm, A_2_ is the absorbance measured at 734 nm in the blank control group, and A_3_ is the absorbance measured at 734 nm in the sample liquid to be tested.

## 3. Results and Discussion

### 3.1. Influence of the Type of Natural Surfactant on the Size of Samples

This section covered the effects of four organic surfactants (Alkyl glucoside, tea saponin, peanut protein isolate, and sucrose fatty acid ester) on the size of TUP. Surfactants were successfully added to prevent the aggregation and proliferation of tiny molecules [17]. The findings indicate that RTP has the largest size, reaching 50 μm. By employing alkyl glycosides as surfactants in the APTUP method, the lowest TUP was produced. Additionally, C alkyl glycoside was the surfactant that had the most impact on TUP size out of the four. D: Isolated peanut protein F: sucrose ester, E: tea saponin. [18]

The sugar component and the hydrophobic alkyl chain determine the bidirectional liquid crystal characteristics of alkyl glycosides [19]. As a result, in addition to the usual lyotropic phase, these amphiphilic surfactants exhibit interesting phase behavior, such as generating various thermotropic phases [20]. Alkyl glycosides, according to Noraini Ahmad et al., are suited as nanoemulsion stabilizers and future drug delivery methods because they promote the creation of tiny nanomolecules [21]. We anticipated that alkyl glycoside molecules would self-assemble in water to create micellar structures, which would aid in wrapping tangeretin molecules and forming uniform and stable ultrafine particles in water.

Alkyl polyglycoside (APG) is a novel kind of green surfactant with excellent interfacial activity, emulsification ability, and foaming performance [22]. It has excellent properties in the APTUP process. When making ultrafine powder with other surfactants, many bubbles will be created, which will make it difficult to make TUP. As a result, we decided that alkyl glycoside would be the ideal surfactant for further research.

### 3.2. Plackett–Burman Design for Particle Size Optimization

According to Isaac’s research, variable optimization was previously primarily based on the “single factor variable analysis” method [23]. However, this method was slow to conduct numerous experiments, lacked statistical analysis of the influence of all determinants, and was unable to explain the interaction between independent factors. PBD factor designs are currently effective and economical and can identify important factors with less research, they can be used as screening designs [24]. Therefore, the goal of this paper is to optimize the operation process by minimizing model errors and reducing the number of experiments run while utilizing mathematical models. In this part, Plackett–Burman Design (Design expert 8.01) was used to study the effects of five factors on TUP particle size. The relevant results are shown in Table 1, and the optimization model equation established by PBD is:

Y = 1183.33 − 107.17A − 195.50B − 133.00C + 304.00D + 58.67E(3)

In Formula (3), A: homogenate speed (r/min), B: surfactant (%), C: rotor diameter (mm), D: concentration (mg/mL), E: liquid–liquid ratio, Y: nano-tangeretin particles size (nm).

The correlation coefficient R^2^ = 0.9301 indicates that the equation can effectively describe the relationship between factors and response values [25], and the significance of each parameter is expressed by *t* and *p* values. The *t*-value corresponding to the positive and negative signs in Equation 3 represents the degree of effect of variables on the response value. A positive value indicates that the factor is positively correlated with the response value, which means that increasing the factor value will increase the response value; a negative value indicates that the factor is negatively correlated with the response value, which means that increasing the factor value will decrease the response value. The D coefficient is positive, indicating that it has the largest impact on *Y* and is positively connected with it. As the D value grows, so does the *Y* value. The coefficients of B and C, on the other hand, are negative, and the impact on *Y* comes in second. The impact of B and C on the *Y* value is inversely proportional. The greater the values of B and C are, the lower the value of *Y*. Therefore, by adjusting the values of these factors, the TUP size can be effectively controlled.

The addition of surfactant can significantly change the electrostatic field forces between the particles, thus changing the repulsive forces between the ultrafine particles and affecting the particle size. In addition, the rotor diameter changes the particle diameter by increasing the contact strength between the solvent and the antisolvent by enhancing the homogenization range. The probability of particle collision decreases with decreasing solution concentration, while an excessive solution concentration leads to particle adhesion, which prevents the formation of ultrafine particles. Therefore, based on the results of the exploratory test, the three PBD optimization factors listed above were determined.

According to Wei et al.’s study, a Pareto diagram was used to rank the priority of components [24], and B (surfactant ratio), C (rotor diameter), and D (solution concentration) had a substantial influence on TUP. The other factors (homogeneous velocity and liquid–liquid ratio) had no influence on TUPAP size. Higher solution concentrations and large amounts of surfactant added in the process of preparing ultrafine particles may result in sufficient collision and precipitation effects in the solution system, resulting in the formation of strong bubbles and cavitation, and these bubbles will cause stronger shock wave and shear force in the solution–antisolvent system, crushing small molecules instantly. As a result, the powder particle size is lowered [26]. A solution concentration that is too low, on the other hand, results in inadequate collision. As a result, BBD must further tune the solution concentration, surfactant ratio, and rotor diameter to provide the best preparation conditions. The importance of these elements is determined by the influence of the particle size obtained and the cost of the procedure. According to the results, the ratio of D (solution concentration) is 52.22%, the ratio of B (surfactant ratio) is 21.59%, and the ratio of C (rotor diameter) is 9.99%, indicating that the linear order of affecting variables is D > B > C > A > E [8].

### 3.3. Particle Size Optimization by Response Surface

The response surface BBD was employed to improve the outcomes of PBD even further. In this section, of the experiment, three variables were employed to explore TUP size: A–concentration, B–surfactant ratio, and C–rotor diameter. The BBD method produced 17 randomized trials to optimize the minimum size of TUP, consisting of two components (BBD and variance analysis), where *p* ≤ 0.0001 is significant (as indicated in Table 2); *R*^2^ was 0.9778; the adjusted *R*^2^ was 0.9492; *C.V.* was 5.12%, the lack of fit *p*-value of 0.2295 (>0.05) was not significant, and the model was significant (<0.0001); therefore, the model has good adaptability in this experiment. The aforementioned statistics demonstrate that the response surface model can precisely forecast the APTUP process produced by the data. The principal components were significant, as indicated by the concentration; however, the influence of the C–rotor diameter was not significant, as indicated by the *p* values of the surfactant ratios being less than 0.05.

The model equation of fitted data is:

Y = 635.17 + 64.18A − 55.18B + 14.27C + 62.96AB + 231.77AC + 100.12BC + 59.76A^2^ + 10.48B^2^ + 141.90C^2^ (4)

where A: solution concentration (mg/mL), B: surfactant (%), C: rotor diameter (mm), and Y: nano-tangeretin particles size (nm).

**Figure 2:**
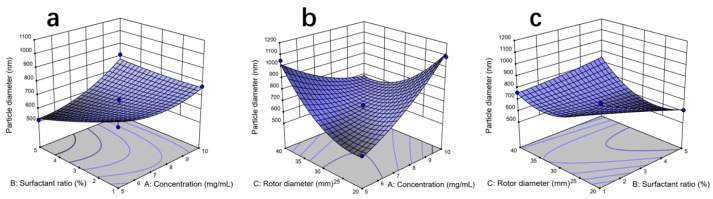
Response surface optimization of powder size prepared by variable factors ((**a**–**c**): significant interaction effect of different factors).

The interplay of several parameters on the size of TUP in the APTUP process is depicted in Figure 2a–c. The high solution concentration (10 mg/mL), which will increase the number of molecular collisions in the antisolvent precipitation process, may be the reason why the concentration change has such a large impact on the size of the powder. Similar to the work of Kitamura et al. [27], an increase in solution temperature does not promote the production of small molecules. The size of the TUP is also significantly influenced by the rotor’s diameter. As shown in Figure 2b, the particle size is larger than 800 nm when the rotor is 20 mm in size, but it is less than 700 nm when the rotor is 40 mm in size. The particle size increases with the rotor diameter, demonstrating a positive correlation between rotor size and particle size. When the rotor diameter decreases to 20 mm, the particle size also significantly decreases. A similar trend is observed with changes in solution concentration: as the concentration of the naringin solution increases, the particle size increases; conversely, a lower concentration results in a marked reduction in particle size. This finding aligns with the study by Valentina Prosapio et al., who prepared folic acid–PVP nanocomposite particles using a supercritical antisolvent method. In their process, reducing the concentration of the DMSO solution led to a smaller average particle size of the folic acid–PVP particles, which is consistent with our results [28].

**Figure 3:**
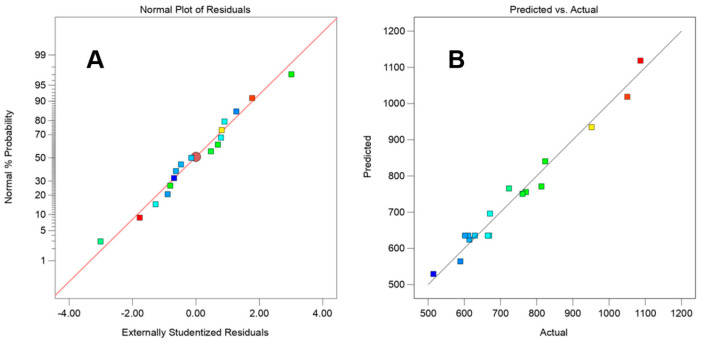
Residual analysis (**A**): normal distribution of residuals, (**B**): predicted and actual distribution.

Through the examination of the residuals’ impact, we can also assess the model’s prediction performance. The residuals in Figure 3A’s normal probability distribution are near a straight line, demonstrating that they follow the rule of a normal distribution and that the fitting effect is satisfactory. In Figure 3B, the distribution of the experimental values and the projected values is likewise very close to a straight line, demonstrating the fitting model’s high adaptability. The BBD response surface model equation prediction was used to optimize the experiment, and the following ideal circumstances were found: The rotor diameter is 22.53 mm, the concentration is 5.23 mg/mL, the surfactant is 4.72%, and the minimum particle size is 407.89 nm. The aforementioned process conditions are modified taking into account the equipment and parameter settings as well as the practicality of the actual operation; the results could be as follows: rotor diameter is 20 mm, the concentration is 5.23 mg/mL, and 4.72% surfactant has been added. After three times of the experimental verification under these conditions, the resulting particle size was about 428.73 ± 30.25 nm. This was marginally higher than the expected value, suggesting that the enhanced extraction method had strong repeatability and that the prediction findings of the aforementioned regression model were more accurate.

### 3.4. The Microscopic Morphology of the Samples

#### 3.4.1. Morphological Observation of Raw and Nano-Tangeretin Powders

**Figure 4:**
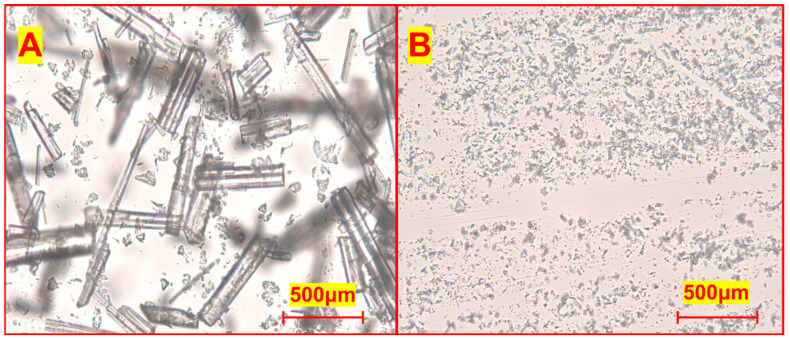
(**A**) raw tangeretin powder and (**B**) nano-tangeretin particles.

According to Figure 4A, the RTP are not homogeneous and exhibit large solids with rectangular outlining, which may be the primary cause of the raw powder’s low water solubility. Figure 4B demonstrates that the ultrafine particles have a modest particle size and are generally consistent in size. It is hypothesized that the solubility may also be enhanced by the uniform distribution of the ultrafine particles and the rise in specific surface area under the microscope observation conditions of the same ratio.

#### 3.4.2. Influence of Different Natural Surfactants on Powder Morphology

**Figure 5:**
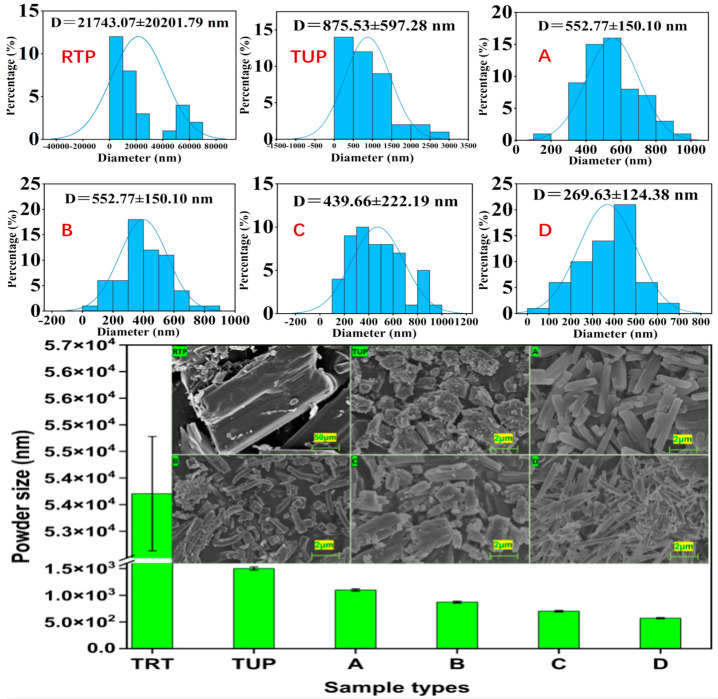
Influence of natural surfactants on powder morphology (RTP: raw tangeretin powder, TUP: nano-tangeretin particles without surfactants, **A**: nano-tangeretin particles with alkyl glucoside, **B**: nano-tangeretin particles with tea saponin, **C**: nano-tangeretin particles with peanut protein isolate, and **D**: nano-tangeretin particles with sucrose fatty acid ester), and DLS analysis.

The microstructure and distribution of particle sizes in RTP and TUP were examined using a scanning electron microscope (Hitachi jsm7500 F, Osaka, Japan); according to Figure 5A, the RTP are not homogeneous and exhibit large solids with rectangular outlining, which may be the primary cause of the raw powder’s low water solubility. Figure 5B demonstrates that the ultrafine particles have a modest particle size and are generally consistent in size. It is hypothesized that the solubility may also be enhanced by the uniform distribution of the ultrafine particles and the rise in specific surface area under the microscope observation conditions of the same ratio. Figure 5A firstly illustrates the nonuniformity of the RTP sample’s particles, which include a big solid with a rectangular profile and larger-than-100 μm particle size that may be the primary feature of the powdered raw material. Particle size reduction and reasonably uniform particle size distribution are observed in TUP samples generated using varying solvents (Figure 5A–D), indicating regular shape properties. Long powder strips with a particle size of around 700 × 2000 nm make up the nano-tangeretin particles containing sucrose fatty acid ester (Figure 5A). The powder’s surface area ratio greatly increases as compared to the RTP. The combination of nano-tangeretin particles with peanut protein (Figure 5B) and tea saponin (Figure 5C) with irregular particle sizes, the isolate’s particle size distribution range is comparatively wide (the particle size was not uniform). Scanning electron microscopy showed that the TUP containing alkyl glucoside was the best result (Figure 5D) as it has the maximum number of ultrafine particles and the smallest particle size among all solvent systems. and DLS research further substantiates this claim. Liu et al. used zein binding tea saponin as a carrier to create bound, encapsulated protein nanoparticles. Additionally, the protein nanoparticles’ solubility was enhanced [29]. Tea saponin increased the compound’s wettability in the solvent system and gave the tiny molecules in the solution system low cost and strong dispersion. As a result, it is frequently employed in the field of emulsifiers to aid in solubility and the generation of nanoparticles [30]. Zhao et al. used peanut protein isolate amyloid-like fibers as stabilizers for high internal phase Pickering emulsions [31]. There are few relevant reports about sugar-based amphiphiles such as alkyl glucoside and sucrose fatty acid ester, which were regarded as a biocompatible, eco-friendly, and milder PEG-free surfactants were widely applied in high-end cosmetic and pharmaceutic [32]. Compared to other surfactants, alkyl glycosides offer a significant advantage over non-ionic surfactants; sugar-derived surfactants show the most promise because of their pH neutral and ion-insensitive nature. The use of sugar ester in high temperature treatment at non-neutral pH is not recommended due to the ester bond’s chemical instability. Conversely, glycosides offer superior stability while preserving the necessary biodegradation characteristics. Regarding the size distribution patterns, we agree that each surfactant influenced the nucleation and growth process differently. This is likely due to the molecular structure and self-assembly behavior of each surfactant. For instance, alkyl glucosides form uniform micelles that encapsulate tangeretin effectively, leading to a narrow distribution (400–500 nm). In contrast, protein-based surfactants such as peanut protein isolate have broader polydispersity due to variable hydrophobic domains.

### 3.5. The Crystal Morphologies of Samples Were Analyzed by X-Ray Diffraction

**Figure 6:**
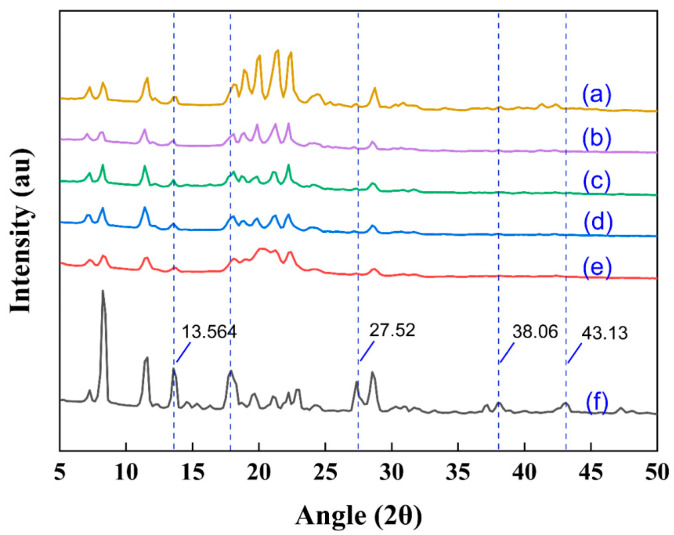
Crystal morphology of the samples (a: TUP without natural surfactants, b: with tea saponin, c: with peanut protein isolate, d: with sucrose fatty acid ester, e: with alkyl glucoside, and f: raw tangeretin powder).

Figure 6a–e show the TUP samples prepared by the antisolvent precipitation method, while Figure 6f presents the unprocessed raw powder sample (RTP). In the X-ray diffraction (XRD) analysis, the RTP sample exhibited stronger diffraction peak intensities and larger crystal particle sizes compared to the TUP samples, although the overall diffraction trends among all samples remained similar. Notably, the RTP sample displayed enhanced peak intensities at 13.56°, 27.52°, 38.06°, and 43.13°, with stable peak widths (Figure 6f), indicating that the RTP exists in a crystalline state. According to the study by G. Sodeifian et al., a decrease in crystallinity typically results in reduced XRD peak intensity, which is usually associated with smaller particle sizes and may lead to the formation of amorphous structures during the natural surfactant-assisted antisolvent precipitation process. In Figure 6a–e, the crystallinity of the TUP samples shows varying reduction. The observed decrease in diffraction peak intensity may be attributed to the disruption of the crystal structure during processing, leading to partially amorphous characteristics. The surface structures of these TUP samples were modified using different additives, which might have left residual compounds in the natural surfactants system and influenced the crystallization. A broad diffuse peak in the range of 10° to 30° was observed in the XRD patterns, suggesting that different surfactants could lead to variations in both the pattern and intensity of the diffraction peaks. Valenzuela and Rodriguez-Llamazares (2016) [16] proposed a method for crystallinity analysis, which involves first smoothing the XRD curve, then distinguishing between crystalline and amorphous regions, and finally calculating the degree of crystallinity. Applying this method, it was found that the crystallinity of the five TUP samples was significantly reduced, likely due to a decrease in particle size.

### 3.6. Infrared Spectrum Scanning of Samples Structure

**Figure 7:**
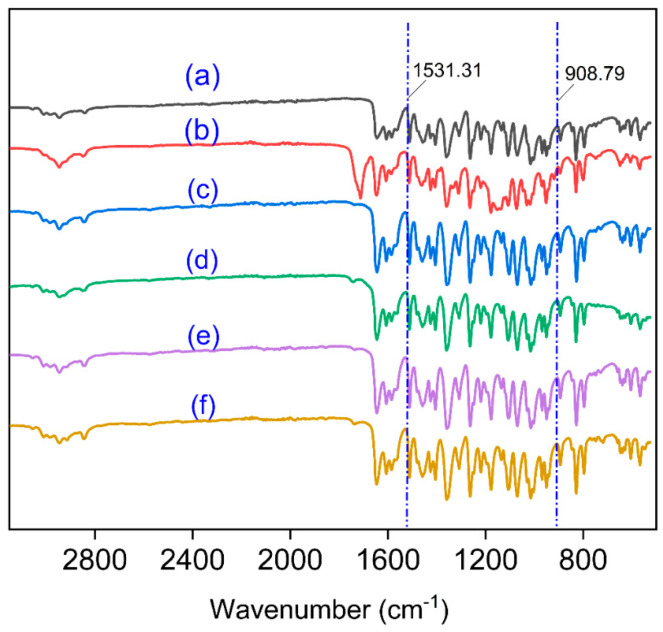
Infrared spectrum data of the samples (a: raw tangeretin powder, b: TUP, c: nano-tangeretin particles with tea saponin, d: nano-tangeretin particles with peanut protein isolate, e: nano-tangeretin particles with sucrose fatty acid ester, and f: nano-tangeretin particles with alkyl glucoside).

The 2 mg tangeretin sample powders and 190 mg KBr powders were carefully weighed; after a good mixing, they become more uniform. We put the mixed powder in a drying oven for 2 min, crushed in the same direction, and then formed into tablets. The findings of a research that used 400–5000 cm^−1^ wavelength infrared scanning was documented. The tangeretin sample’s infrared spectrum data are displayed in Figure 7a–f. To begin with, nearly every sample (RTP, TUP) displayed the same tensile trend. The sample prepared without natural surfactant (Figure 7b) was more significantly stretched forward (1700–1750 cm^−1^) in the TUP sample. The remaining samples all displayed the same stretching tendency and remarkably comparable patterns and trends, suggesting that TUP stretching of the surfactant preparation may be the origin of these minor variations in IR spectra. Consequently, in the samples made using this technique, the chemical structures of RTP and TUP were unchanged. The KBr pellet method may disrupt weak intermolecular interactions within the sample. Although ATR instrumentation was not available during the course of this study, ATR-FTIR analysis will be introduced in future work to further validate the conclusions of this study.

### 3.7. Thermodynamic Analysis of Samples

**Figure 8:**
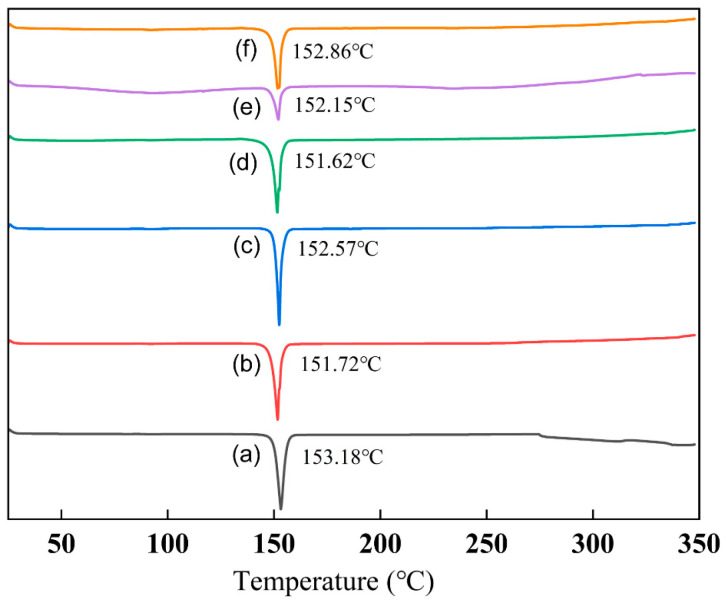
The thermograms of tangeretin samples prepared under different conditions obtained from DSC analysis. (a: raw tangeretin powder, b: TUP, c: nano-tangeretin particles with tea saponin, d: nano-tangeretin particles with peanut protein isolate, e: nano-tangeretin particles with sucrose fatty acid ester, and f: nano-tangeretin particles with alkyl glucoside).

To investigate the thermal behavior of the tangeretin samples before and after processing, and to assess potential changes in their physicochemical properties, a differential scanning calorimetry (DSC) analysis was conducted. As shown in Figure 8, all samples exhibited similar thermal profiles. Upon gradual heating, distinct exothermic peaks appeared at nearly the same temperature range for all samples—specifically at 153.18 °C (a), 151.72 °C (b), 152.57 °C (c), 151.62 °C (d), 152.15 °C (e), and 152.86 °C (f). This consistent melting behavior suggests that the chemical characteristics of tangeretin remained largely unchanged after processing (as shown in Figure 8). Furthermore, the similarity in peak patterns indicates that both the raw powder and the UPN samples possess low crystallinity and likely exist in an amorphous or poorly defined structural form. The thermal behavior of tangeretin is significantly influenced by its interaction with surfactants. Differential Scanning Calorimetry (DSC) results show that strong surfactant–tangeretin interactions can lead to a reduction or disappearance of sharp melting peaks, indicating improved stabilization and possible amorphization [33]. In particular, formulations containing sucrose fatty acid esters exhibited weakened endothermic peaks, suggesting strong binding between the surfactant and tangeretin molecules. This interaction not only enhances thermal stability but also potentially improves solubility and bioavailability, highlighting the importance of surfactant selection in formulation design.

### 3.8. Comparison of Antioxidant Properties of Samples Before and After Preparation

**Figure 9:**
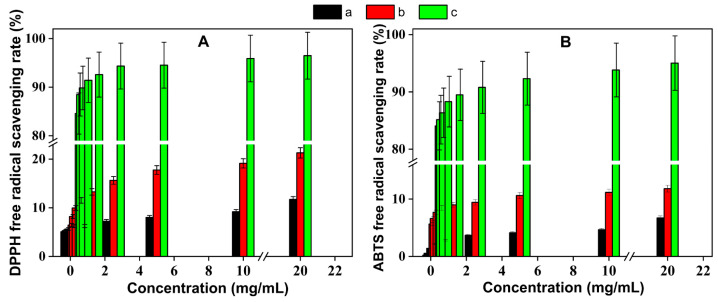
Free radical scavenging effect of tangeretin samples ((**A**): DPPH, (**B**): ABTS, a: RTP, b: TUP, c: BHT).

The antioxidant data and Figure 9 were plotted through origin (Version 11.0) was developed by OriginLab Corporation, located in Northampton, MA, USA and then discussed. When the sample solution concentration was 20 mg/mL, the free radical scavenging rate of TUP increased to 21.29%, whereas that of RTP was only 11.72%. The aforementioned findings showed a favorable correlation between the sample concentration and the DPPH free radicals’ ability to scavenge oxygen. The capacity of TUP to scavenge DPPH free radicals was greater than that of RTP because its scavenging activity was 1.82 times that of RTP (see Figure 9A). RTP and TUP had scavenging rates of 6.72% and 11.81%, respectively, for the ABTS free radical. TUP contains 1.75 times more ABTS free radical scavenging power than RTP, and the produced sample has a greater antioxidant effect (Figure 9B). In conclusion, the produced samples had a superior antioxidant effect and exhibited a concentration–dose dependency that was consistent with the findings of the study by Kakran et al. [34]. As a result, TUP now has many more antioxidant qualities, making it appropriate for the creation of natural antioxidant products. TUP produced by TUPAP technique had a considerably greater scavenging ability for DPPH and ABTS free radicals than RTP aqueous solution, as compared to RTP. A significant correlation was seen between the capacity to scavenge hydroxyl radicals and the rise in sample concentration. According to Patel et al. [35], the compound’s reducing capacity and antioxidant capacity were directly correlated, and the solution system’s absorbance rose as a result. RTP has a relatively low antioxidant capacity, which could be related to how poorly it can provide electrons. It is hypothesized that RTP’s low antioxidant capability results from its limited water solubility. As a result, the solution’s concentration, and the absorbance of tangeretin samples were connected. Although RTP and TUP lack phenolic hydroxyl groups, IC_30_ analysis was conducted to quantify their relative radical scavenging capacities. As shown in Table S1, both RTP and TUP exhibited significantly weaker antioxidant activity compared to BHT. However, TUP showed a moderately lower IC_30_ value than RTP in both DPPH and ABTS assays, indicating a slightly enhanced radical scavenging ability, possibly due to surface modifications during processing.

The solution absorbance of TUP is greater than that of RTP because it has a smaller particle size than the original daidzein and could be more water soluble [36]. The absorbance at 734 nm and quick reaction time of the ABTS free radical scavenging test are noteworthy. As a result, it may also be utilized as a gauge for a compound’s antioxidant activity. Both treated and unprocessed samples exhibited a dose-dependent scavenging action against ABTS free radicals. The ABTS free radical scavenging ability of the samples decreased in the following order: BHT > TUP > RTP. Treated TUP exhibited significant potential as an ABTS radical scavenger, which may be attributed to its solubility—since the extent of TUP dissolution appears to influence its capacity to scavenge both DPPH and ABTS radicals, which is similar to the results of Liu et al. [37]. There is potential use for micronized TUP samples as an antioxidant in the food and pharmaceutical sectors.

## 4. Conclusions

Technology for solvent–antisolvent precipitation is a useful ultrafine technology. Natural surfactants are used in this procedure to help with the preparation of ultrafine powder, and they not only improve the production process’ cleanliness but also have more profound impacts. Alkyl glycosides were the best natural surfactants chosen for this study, and the five parameters of concentration (mg/mL) and liquid–liquid ratio (mL/mL) were adjusted. The homogenization speed (r/min), surfactant ratio (%), rotor diameter (mm), and surfactant ratio (%) were also optimized. Based on this, BBD was able to produce TUP with an average particle size of 428.73 ± 30.25 nm. The samples before and after preparation revealed no chemical changes, according to SEM, XRD, and FTIR; however, the particle size of TUP dramatically decreased. Alkyl glycoside is the best option for a solvent–antisolvent system since it is a natural surfactant. Furthermore, the preliminary evaluation of antioxidant properties of TUP was almost two times greater than that of RTP, demonstrating the efficacy of the tangeretin product produced using the APTUP method with natural surfactants added. This method also offers effective technical support for the clean production of natural compounds and is better suited for the processing of nontoxic green foods and nature antioxidant products.

## Figures and Tables

**Table 1:** Plackett–Burman design results for variables.

No.	A: Homogenate Speed (r/min)	B: Surfactant Ratio (%)	C: Rotor Diameter (mm)	D: Conentration (mg/mL)	E: Liquid–Liquid Ratio (mL/mL)	Particle Size (nm)
1	600	1.00	20.00	5.00	10.00	1220
2	600	5.00	20.00	5.00	40.00	990
3	1200	5.00	40.00	5.00	40.00	412
4	600	1.00	40.00	10.00	10.00	1646
5	1200	1.00	40.00	10.00	40.00	1399
6	600	5.00	40.00	10.00	40.00	1481
7	1200	1.00	40.00	5.00	10.00	838
8	600	5.00	40.00	5.00	10.00	526
9	1200	5.00	20.00	10.00	10.00	1430
10	1200	1.00	20.00	5.00	40.00	1290
11	1200	5.00	20.00	10.00	10.00	1088
12	600	1.00	20.00	10.00	40.00	1880
**ANOVA**	**Sum of squares**	**Degree of freedom**	**Mean square**	**F-value**	***p*-value**	**Significance level**
Model	1.959 × 10^6^	5	3.918 × 10^5^	16.57	0.0019	**
Residual	1.418 × 10^5^	6	23,638.67			
Cor Total	2.101 × 10^6^	11				
**Regression equation**	**Coefficient**	**Standard Error**	**F-Value**	***p*-Value**	**Significance level**	
A	−107.17	44.38	5.83	0.0522	NS	
B	−195.50	44.38	19.40	0.0045	**	
C	−133.00	44.38	8.98	0.0241	*	
D	304.00	44.38	46.91	0.0005	**	
E	58.67	44.38	1.75	0.2344	NS	

*: Significant at *p* ≤ 0.05; **: significant at *p* ≤ 0.01; NS: not significant.

**Table 2:** Box–Behnken design with experimental value for total size of powder (nm), analysis of variance (ANOVA) for response surface quadratic model and fit statistics for response values.

Run	BBD				ANOVA								
	A (mg/mL)	B (%)	C (mm)	Y (nm)	Source	SS	DF	Mean Square		F-Value	*p*-Value		
1	10	5	30	813.21	Model	4.356 × 10^5^	9	48,400.54		34.23	<0.0001 **		
2	10	3	20	1086.68	A-Concentration	32,951.30	1	32,951.30		23.31	0.0019		
3	7.5	3	30	629.38	B-Surfactant ratio	24,355.35	1	24,355.35		17.23	0.0043		
4	7.5	1	20	951.89	C-Rotor diameter	1629.06	1	1629.06		1.15	0.3187		
5	7.5	3	30	668.65	AB	15,854.59	1	15,854.59		11.21	0.0123		
6	7.5	5	40	823.45	AC	2.149 × 10^5^	1	2.149 × 10^5^		151.97	<0.0001 **		
7	5	5	30	514.59	BC	40,096.06	1	40,096.06		28.36	0.0011		
8	10	1	30	770.32	A^2^	15,035.74	1	15,035.74		10.63	0.0138		
9	7.5	3	30	664.89	B^2^	462.69	1	462.69		0.33	0.5852		
10	7.5	5	20	613.97	C^2^	84775.84	1	84775.84		59.96	0.0001 **		
11	7.5	1	40	760.89	Residual	9897.38	7	1413.91					
12	10	3	40	670.98	Lack of Fit	6171.97	3	2057.32		2.21	0.2295		
13	5	1	30	723.53	Pure Error	3725.41	4	931.35					
14	7.5	3	30	601.98	Cor Total	4.455 × 10^5^	16						
15	5	3	40	1050.51	Credibility analysis of the regression equations								
16	7.5	3	30	610.96	Index mark	Standard deviation	Mean	CV%	Press	R^2^	Adjust R^2^	Predicted R^2^	Adequacy precision
17	5	3	20	539.13	Y	37.6	735	5.12	76.75	0.9778	0.9492	0.7653	20.645

A: solution concentration (mg/mL), B: surfactant ratio (%), C: rotor diameter (mm), Y: nano-tangeretin particles size (nm), DF: degree of freedom, SS: sum of squares. **: Significant.

## Data Availability

Data are contained within the article and Supplementary Materials.

## Supplementary Materials

The following supporting information can be downloaded at: https://www.mdpi.com/article/10.3390/nano15110791/s1, Table S1: IC₃₀ values of RTP, TUP, and BHT in DPPH and ABTS radical scavenging assays. The data were expressed as mean ± standard deviation (n = 3).

## Abbreviations

RTP	Raw tangeretin powder
PBD	Plackett–Burman design
BBD	Box–Behnken design
DMSO	Dimethyl sulfoxide
