# Supplementary material for: Optimization of Nano-Tangeretin Recrystallization via Natural Surfactants in the Antisolvent Precipitation Process: Physicochemical Characterization and Antioxidant Activity
Source: Nanomaterials (Basel). 2025 May 24;15(11):791. doi: 10.3390/nano15110791 (PMC12157902; doi:10.3390/nano15110791)
Supplement: Supplementary file 1 [file nanomaterials-15-00791-s001.zip › nanomaterials-3627630-supplementary.pdf]

Table S1. IC_{30} values of RTP, TUP, and BHT in DPPH and ABTS radical scavenging assays. The data were expressed as mean±standard deviation (n = 3).

Samples	DPPH IC_{30} (μg/mL)	ABTS IC_{30} (μg/mL)
RTP	185.2±4.3	162.7±5.1
TUP	132.8±3.7	118.4±4.8
BHT	41.6±1.2	36.9±1.5
